# Pharmacoeconomic analysis of prostaglandin and prostamide therapy for patients with glaucoma or ocular hypertension

**DOI:** 10.1186/1471-2415-7-16

**Published:** 2007-09-27

**Authors:** Ronald EP Frenkel, Max Frenkel, Allison Toler

**Affiliations:** 1 Bascom Palmer Eye Institute, University of Miami School of Medicine, Miami, Florida, USA; 2 Eye Research Foundation, Stuart, Florida, USA; 3 East Florida Eye Institute, Stuart, Florida, USA

## Abstract

**Background:**

To determine monthly cost and cost effectiveness of bilateral prostaglandin/prostamide therapy for lowering intraocular pressure (IOP) in patients taking bimatoprost 0.03% (Lumigan^®^, Allergan, Inc.), latanoprost 0.005% (Xalatan^®^, Pfizer, Inc.), or travoprost 0.004% (Travatan^®^, Alcon Laboratories, Inc.).

**Methods:**

Drops in five new 2.5-mL bottles were counted and then averaged for each drug. Average retail price was determined by surveys of pharmacies. Drop count, average retail price, average wholesale price, and IOP reduction data were used to compute annual cost, and cost effectiveness (annual cost-per-mm Hg of IOP reduction) of the three drugs.

**Results:**

Drops per 2.5-mL bottle averaged 113 for bimatoprost 0.03%, 84 for latanoprost 0.005%, and 83 for travoprost 0.004%. Average retail cost (2005) per bottle was $69.99 for bimatoprost 0.03%, $61.69 for latanoprost 0.005%, and $66.37 for travoprost 0.004%. The monthly retail cost of bilateral therapy was $37.92 for bimatoprost 0.03%, $44.75 for latanoprost 0.005%, and $49.25 for travoprost 0.004%. Cost effectiveness ranges were $57 to $65 per mm Hg reduction in IOP per year for bimatoprost, 0.03%, $67 to $90 per mm Hg for latanoprost 0.005%, and $74 to $84 per mm Hg for travoprost 0.004%.

**Conclusion:**

Bimatoprost 0.03% had the lowest monthly and annual costs and the greatest cost effectiveness for lowering IOP compared with latanoprost 0.005% and travoprost 0.004%.

## Background

The prostamide bimatoprost and the prostaglandin analogues latanoprost and travoprost are ocular hypotensive lipids that are indicated for lowering intraocular pressure (IOP) in patients with glaucoma or ocular hypertension. The efficacy of bimatoprost 0.03% (Lumigan^®^, Allergan, Inc. Irvine, CA), latanoprost 0.005% (Xalatan^®^, Pfizer, Inc., New York, NY), and travoprost 0.004% (Travatan^®^, Alcon Laboratories, Inc. Ft. Worth, TX) was superior to timolol for lowering IOP in these patient populations [1-3]. One [4] of two recent studies [4,5] demonstrated that bimatoprost is statistically significantly more effective than latanoprost in lowering IOP.

The costs (direct and indirect) of pharmaceuticals to patients have become an important national issue. Managed care organizations utilize pharmacoeconomic data such as cost minimization and cost effectiveness when deciding which drugs to include in their formulary. *Cost minimization *involves choosing a drug based on direct costs, whereas *cost effectiveness *is determined by dividing the direct cost of the drug by its efficacy, defined in this analysis as mm Hg of IOP reduction. The relationship between decisions based on cost minimization and those based on cost effectiveness analyses is shown in Table 1.

**Table 1:** Cost effectiveness example calculation

IOP reduction	Direct costs	Cost effectiveness calculations	
Decision based on **cost minimization**:			
Equal (10 mm Hg)	Different ($500/yr vs. $400/yr)	Drug A: $500/yr; 10 mm Hg reduction = $50 per mm Hg reduction	Drug B: $400/yr; 10 mm Hg reduction = $40 per mm Hg reduction
Decision based on **cost effectiveness**:			
Different (10 mm Hg vs. 5 mm Hg)	Different ($500/yr vs. $400/yr)	Drug C: $500/yr; 10 mm Hg reduction = $50 per mm Hg reduction	Drug D: $400/yr; 5 mm Hg reduction = $80 per mm Hg reduction

IOP = intraocular pressure When the formulary inclusion decision process is based on cost minimization, Drug B is chosen over Drug A based on the difference in direct costs. When the decision is based on cost effectiveness, Drug C is chosen over Drug D in spite of the higher direct cost of Drug C, because Drug C is more cost effective.

If the dispensable shelf life of an ophthalmic preparation requires its disposal before the entire bottle of drug solution has been used [6], cost effectiveness can be affected. Bimatoprost has a shelf life of 2 years [7], whereas latanoprost must be used within 6 weeks of opening [8]. No shelf life limitation is indicated in the current prescribing information for travoprost [9]. Limited dispensable shelf life is one reason for incomplete use of issued medication, which may affect the number of bottles required for treatment over time.

The objective of this study was to compare monthly costs (based on retail and wholesale prices) and the cost effectiveness of bilateral treatment with bimatoprost 0.03%, latanoprost 0.005%, and travoprost 0.004% for reduction of IOP in patients with glaucoma or ocular hypertension.

## Methods

This prospective pharmacoeconomic study evaluated the direct cost and cost effectiveness of prostaglandin/prostamide therapy for reduction of IOP in patients with glaucoma or ocular hypertension. The drugs studied were bimatoprost 0.03% (Lumigan^®^, Allergan, Inc. Irvine, CA), latanoprost 0.005% (Xalatan^®^, Pfizer, Inc., New York, NY), and travoprost 0.004% (Travatan^®^, Alcon Laboratories, Inc. Ft. Worth, TX).

Retail prices of the 3 drugs were obtained from 7 pharmacies representing both independent (Park Pharmacy) and chain (Walgreens, Wal-Mart, Winn Dixie, K-Mart, Target, and DrugStore.Com) businesses. The pharmacies were located in Stuart, FL, except for Drugstore.com which is an online pharmacy based in Bellevue, WA. All pharmacies were chosen prior to their pricing being known and no pharmacies were dropped from the study. Prices were obtained during 3 different time periods: 1) during the fall of 2001, when a drop-count study was also conducted, 2) during February 2003, and 3) during May 2005. Prices were checked twice in 2 weeks and immediately recorded.

The contents of 5 new bottles (marked as containing 2.5 mL) of each drug (bimatoprost 0.03%, latanoprost 0.005%, and travoprost 0.004%) were measured in terms of drops per bottle by a procedure designed to mimic actual medication use by patients. Drops were counted while the contents were at room temperature. Each bottle was inverted to a vertical position and squeezed until 1 drop came out. The bottle was then placed upright. This procedure was repeated until the bottle was emptied. The same person dispensed all drops for all 3 drugs and was masked to the purpose of the study and the identity of the drugs. Because a secondary observer might be more likely to miss seeing a drop, the same person that squeezed the bottles also counted the drops. The average of the values for the 5 bottles was used in further analyses.

Cost-minimization analysis was conducted using the average retail price, determined from the pharmacy survey, for 1 bottle of drug from May 2005 and also with the average wholesale price (AWP) from the same year. The number of days per bottle was calculated by dividing the number of drops per bottle by 2 (drops per day), based on treatment of both eyes once per day. Annual usage (bottles per year) was calculated by dividing 365 (days per year) by the number of days per bottle. Annual cost is the bottle cost times the annual usage, and the monthly cost is the annual cost divided by 12 (months per year). An additional analysis was made with the assumption that patients occasionally misdirect their medication at instillation and require 2 extra drops per week. For this calculation, annual usage was based on daily, bilateral dosing plus 2 extra drops per week.

Annual usage, annual cost, and monthly cost were also considered with regard to drug dispensable shelf life. The effect on treatment costs was found by comparing the shelf life, based on prescribing information, with the number of treatment days per bottle.

Cost effectiveness was determined by dividing the drug cost (2005 AWP) by the drug's efficacy. In this study, efficacy is defined as the degree of IOP reduction (mm Hg), taken from the package insert for each drug [7-9]. Cost effectiveness data for the three drugs in this study are expressed as ranges calculated from the ranges of IOP lowering reported in the respective package inserts.

## Results

The results from a survey of 7 different pharmacies regarding the costs of 2.5 and 5-mL bottles of bimatoprost 0.03%, a 2.5-mL bottle of latanoprost 0.005%, and a 2.5-mL bottle of travoprost 0.004% in the fall of 2001 appear in Table 2. Drops from the 5-mL bottles were not counted, nor were the costs for these bottles used in any of the calculations; however, these data are presented to provide additional information. The highest average cost was found for bimatoprost 0.03%, followed by travoprost 0.004% and latanoprost 0.005%.

**Table 2:** US retail drug prices, fall 2001

	**Bimatoprost 0.03%, 2.5 mL**	**Bimatoprost 0.03%, 5.0 mL ($ per 2.5 mL)**	**Latanoprost 0.005%, 2.5 mL**	**Travoprost 0.004%, 2.5 mL**
Walgreens	$54.99	$52.50	$53.99	--
Park Pharmacy	$51.95	$50.98	$49.95	$49.95
WalMart	$51.46	$51.89	$40.98	$48.62
Winn Dixie	$44.50	$44.50	$46.00	$47.00
K-Mart	$56.97	$51.99	$49.99	$53.97
Target	$56.39	$51.35	$41.99	$53.69
DrugStore.Com	$50.10	$50.10	$46.90	$47.40
**Average**	**$52.34**	**$50.47**	**$47.11**	**$50.11**

Due to inflation, new retail prices were obtained from the same pharmacies in February 2003 (Table 3) and May 2005 (Table 4). The average retail price increased 19% for bimatoprost 0.03%, 18% for travoprost 0.004%, and 8% for latanoprost 0.005% from 2001 to 2003 (Table 5). Over the next 2 years prices continued to increase. By May 2005, average retail prices for 2.5-mL bottles were $61.69 for latanoprost 0.005% (range $50.68 to $69.95), $66.37 for travoprost 0.004% (range $56.88 to $71.99), and $69.99 for bimatoprost 0.03% (range $57.74 to $74.99). This represented a further increase of 13% for bimatoprost 0.03%, 12% for travoprost 0.004%, and 21% for latanoprost 0.005% from 2003 to 2005.

**Table 3:** US retail drug prices, February 2003

	**Bimatoprost 0.03%, 2.5 mL**	**Bimatoprost 0.03%, 5.0 mL ($ per 2.5 mL)**	**Latanoprost 0.005%, 2.5 mL**	**Travoprost 0.004%, 2.5 mL**
Walgreens	$61.99	$59.00	$56.99	--
Park Pharmacy	$56.90	$55.45	$52.90	$56.90
WalMart	$54.72	$52.73	$41.99	$54.72
Winn Dixie	$84.95	$50.98	$52.95	$71.95
K-Mart	$61.97	$56.99	$52.99	$61.97
Target	$61.64	$48.00	$46.69	$57.84
DrugStore.Com	$52.64	$50.10	$51.55	$50.86
**Average**	**$62.12**	**$53.86**	**$50.87**	**$59.04**

**Table 4:** US retail drug prices, May 2005

	**Bimatoprost 0.03%, 2.5 mL**	**Bimatoprost 0.03%, 5.0 mL ($ per 2.5 mL)**	**Latanoprost 0.005%, 2.5 mL**	**Travoprost 0.004%, 2.5 mL**
Walgreens	$74.99	$71.50	$65.99	$71.99
Park Pharmacy	$68.95	$67.98	$63.98	$64.95
WalMart	$69.32	$63.23	$50.68	$64.88
Winn Dixie	$71.95	$63.48	$69.95	$66.95
K-Mart	$74.97	$69.49	$62.99	$70.97
Target	$71.99	$65.75	$59.99	$67.99
DrugStore.Com	$57.75	$55.12	$58.22	$56.88
**Average**	**$69.99**	**$65.22**	**$61.69**	**$66.37**

**Table 5:** Percentage increase in average retail price

	**Fall 2001 to February 2003**	**February 2003 to May 2005**	**Fall 2001 to May 2005**
**Bimatoprost 0.03%**	19%	13%	34%
**Latanoprost 0.005%**	8%	21%	31%
**Travoprost 0.004%**	18%	12%	32%

The number of drops counted from 2.5 mL bottles of the 3 drugs appears in Table 6. Bottles of bimatoprost 0.03% had the greatest number of drops, followed by latanoprost 0.005%, and then by travoprost 0.004%. The standard deviation of the number of drops per bottle was highest for bimatoprost and lowest for latanoprost.

**Table 6:** Drops per bottle

	**Bimatoprost 0.03%**	**Latanoprost 0.005%**	**Travoprost 0.004%**
**Bottle 1**	118	83	88
**Bottle 2**	130	88	97
**Bottle 3**	100	82	78
**Bottle 4**	103	85	75
**Bottle 5**	115	81	79
**Average ± SD**	**113 +/- 12**	**84 +/- 3**	**83 +/- 9**

Based on average retail prices from 2005, calculations for the number of days per bottle, annual usage, annual cost, monthly cost, bilateral daily cost and cost per drop appear in Table 7 for each of the 3 medications. With bilateral therapy, the retail costs per day for these medications are $1.24 for bimatoprost 0.03%, $1.46 for latanoprost 0.005%, and $1.60 for travoprost 0.004%. The costs were lowest for bimatoprost 0.03% and highest for travoprost 0.004%, by a difference of 30%.

**Table 7:** Cost-minimization analysis of bimatoprost 0.03%, latanoprost 0.005%, and travoprost 0.004%; average retail price

	**Drops per Bottle**	**Days per Bottle**	**Annual Usage (bottles per year)**	Bottle Cost* ($ per bottle)	**Annual Cost**	**Monthly Cost**	**Cost per Day (bilateral therapy)**	**Cost per Drop**
**Bimatoprost 0.03%**	113	56	6.5	$69.99	$455	$37.92	$1.24	$0.62
**Latanoprost 0.005%**	84	42	8.7	$61.69	$537	$44.75	$1.46	$0.73
**Travoprost 0.004%**	83	41	8.9	$66.37	$591	$49.25	$1.60	$0.80

* Average retail price, 2005

Similarly, Table 8 lists the annual and monthly costs of the 3 medications but substitutes AWP from 2005 for average retail price in the calculations. The AWP for one bottle is lowest for latanoprost 0.005% and highest for travoprost 0.004%, with a difference of only 1.5%. However, the annual and monthly costs based on AWP are lowest for bimatoprost 0.03% and highest for travoprost 0.004%, with a difference of 37%.

**Table 8:** Cost-minimization analysis of bimatoprost 0.03%, latanoprost 0.005%, and travoprost 0.004%; average wholesale price

**Drug**	**Drops per Bottle**	**Days per Bottle**	**Annual Usage (bottles per year)**	Bottle Cost* ($ per bottle)	**Annual Cost**	**Monthly Cost**
**Bimatoprost 0.03%**	113	56	6.5	$62.10	$404	$33.67
**Latanoprost 0.005%**	84	42	8.7	$61.29	$533	$44.42
**Travoprost 0.004%**	83	41	8.9	$62.19	$553	$46.08

* Average Wholesale Price, 2005

The results of an analysis that assumes 2 doses missed the eye per week (thus requiring reapplication) appear in Table 9. A total of 834 drops per year are required for any of the 3 drugs. Using this assumption and the average retail price from 2005, the annual and monthly costs are shown.

**Table 9:** Cost-minimization analysis of bimatoprost 0.03%, latanoprost 0.005%, and travoprost 0.004%; assuming 2 drops missed the eye per week

**Drug**	**Drops per Bottle**	**Drops Administered per Year (bilaterally)**	**Drops Missed Hitting the Eye per Year**	**Total Drops per Year**	**Bottles per Year**	Bottle Cost* ($ per bottle)	**Annual Cost**	**Monthly Cost**
**Bimatoprost 0.03%**	113	730	104	834	7.4	$69.99	$518	$43.17
**Latanoprost 0.005%**	84	730	104	834	9.9	$61.69	$611	$50.92
**Travoprost 0.004%**	83	730	104	834	10.0	$66.37	$664	$55.33

*Average retail price, 2005

The shelf lives of bimatoprost 0.03% (2 years) and latanoprost 0.005% (42 days) were not limiting, and were greater than or equal to the number of days per bottle based on bilateral, daily dosing (bimatoprost 0.03% = 56 days, latanoprost 0.005% = 42 days). No limitation of the shelf life for travoprost 0.004% appears in its prescribing information [9]. Thus, when considering drug dispensable shelf life, no corrections were required for calculations of annual usage, annual cost, and monthly cost for any of the 3 medications.

Results of the cost effectiveness analysis appear in Table 10. The annual costs used for these calculations were based on average retail price in 2005 from Table 4. The efficacy ranges were taken from the most recent prescribing information [7-9]. For bimatoprost 0.03%, an annual cost of $455 with an efficacy range of 7 to 8 mm Hg of IOP reduction [7] yielded a range of cost effectiveness from $57 to $65 per 1 mm Hg decrease in IOP. Bimatoprost 0.03% was more cost effective than latanoprost 0.005% and travoprost 0.004%.

**Table 10:** Cost effectiveness analysis of bimatoprost 0.03%, latanoprost 0.005%, and travoprost 0.004%

	**Bimatoprost 0.03%**	**Latanoprost 0.005%**	**Travoprost 0.004%**
Annual Cost*	$455	$537	$591
**IOP Reduction, Lower Value^†^(mm Hg)**	7	6	7
**IOP Reduction, Upper Value^†^(mm Hg)**	8	8	8
**Cost Effectiveness Range ($/mm Hg decrease in IOP)**	$57–$65/mm Hg	$67–$90/mm Hg	$74–$84/mm Hg

IOP = intraocular pressure

*Average retail price, 2005, before adjustment for drug shelf life.

^†^Ranges of IOP reduction efficacy reported in package inserts [7–9].

## Discussion

Bimatoprost 0.03% was the most economical of the 3 prostaglandin/prostamide IOP-lowering drugs evaluated in this study in terms of both cost minimization and cost effectiveness.

The monthly and annual costs of bimatoprost 0.03% were the lowest of the 3 medications in the cost minimization analyses because of the greater number of drops dispensed per bottle, a measurement that mimics actual use of medication by patients. In this study, means of 113, 84, and 83 drops per 2.5 mL bottle of bimatoprost, latanoprost, and travoprost, respectively, were measured. The relative rank of bimatoprost over latanoprost and travoprost is consistent with 3 previous studies which measured 111, 98, and 103 drops [10]; 103, 92, and 98 drops [11]; and 111.0, 94.3, 81.4 drops (vertical position) [12] per 2.5 mL bottle of bimatoprost, latanoprost, and travoprost, respectively. The total volumes of medications dispensed in those studies were 3.3, 3.05, and 3.0 mL [10]; 3.06, 2.98, and 2.92 mL [11]; and 3.17, 3.02, 2.54 mL [12] per 2.5 mL bottle of bimatoprost, latanoprost, and travoprost, respectively.

Bimatoprost remained the most economical whether or not product dispensable shelf life was accounted for, whether average retail prices or average wholesale prices were used in the calculations, or if additional medication was needed due to occasional misdirection of drops during instillation. Overall, the monthly and annual costs of binocular therapy with latanoprost 0.005% and travoprost 0.004% ranged from 18% to 37% more than for bimatoprost 0.03%, depending on the premise of the analysis. The results of another cost comparison of these three drugs by Mick et al. [11] also found bimatoprost to be the least expensive, but the cost differences between the 3 drugs were less. These investigators demonstrated that the annual cost of monocular therapy with bimatoprost was less than with latanoprost, travoprost, or unoprostone. In addition, they found that bimatoprost had the largest percentage of overfill from the labeled volume of 2.5-mL compared with the other three medications.

Overall, prices for these medications increased 31 to 34% from 2001 to 2005, while the consumer price index increased 10% [13]. It is interesting that the disparity between the pharmaceutical price increase and the Consumer Price Index was similar for all three drugs. The reasons for this are beyond the scope of this paper; but clearly, the cost of pharmaceuticals is an important issue today and will likely remain so into the future.

Bimatoprost 0.03% was the most cost effective (i.e., had the lowest cost per mm Hg of IOP reduction) of the three drugs evaluated, a finding consistent with other pharmacoeconomic studies of IOP-lowering medications [14]. Efficacy in this study was based on ranges of IOP reductions published in the prescribing information for each drug, but greater differences in efficacy may be observed among individual patients or in clinical studies. In contrast to the similar IOP reduction ranges documented in the prescribing information for these drugs, Noecker et al. [4] demonstrated that bimatoprost 0.03% had IOP reduction efficacy that was superior to latanoprost 0.005% [7,8]. Wider differences in efficacy may contribute to more disparate cost effectiveness, as shown in analyses based on the number of patients that achieved a target decrease in IOP [15] or that used a patient-weighted average IOP reduction based on IOP lowering reports for the 3 drugs from over 20 different studies [16]. These studies showed that bimatoprost produced a larger percent change in IOP, and was thus more cost effective than latanoprost [15,16] and travoprost [16]. These analyses did not account for differences in the dispensation of the medications, but do suggest that the differences in the cost effectiveness of these 3 drugs may be greater in practice than reported here.

This study was limited by the number of bottles of each drug that were counted. The volume of ophthalmic solution (drops per bottle) could vary considerably with each medication and across medications. Each medication in this study was dispensed from a vertically-positioned bottle, but the angle of instillation has been found to differentially affect drop size and thus the number of days of therapy achieved [12]. Additionally, a small number of pharmacies were surveyed, although most were members of large chains that were felt to be representative retailers to consumers. One local non-chain pharmacy (Park) was chosen to represent that particular segment of the market. We tried to be unbiased in our pharmacy selection; however, it is clear that retail costs to patients with no drug coverage may vary among these and other pharmacies. Furthermore, although patients with drug coverage may be less affected depending on their deductible level, these cost variances may still be consequential.

This study did not address different drug tolerabilities [5] which may or may not cause patients to discontinue therapy, nor did it address the concept of persistency. Parrish et al. [5] found that latanoprost exhibited greater ocular tolerability than bimatoprost or travoprost. Patients using a less tolerable drug might also use artificial tears to increase ocular comfort, which could increase the total cost of therapy. This determination was beyond the scope of this study. Drug discontinuation rates can also affect cost. Greater patient persistency with one medication over another would potentially lower the overall cost of therapy with that drug, taking into account the associated cost of additional physician visits if a patient needs to switch to another drug. Although Reardon et al. [17] showed relatively better persistency with latanoprost, a more recent study showed comparable persistency among latanoprost, bimatoprost, and travoprost, and slightly better adherence with bimatoprost than latanoprost [18]. Even with a distinct between-drug difference in persistency, it is difficult to quantitate the effect of persistency on therapy cost, particularly across different health care delivery settings. Such costs would have to be amortized over variable durations of the patients' therapy. Though such costs may be real, assigning additional costs related to tolerability or persistence would have introduced an inexact variable into this objective quantitative analysis.

## Conclusion

The data provided by this analysis should be useful to those interested in the costs of common glaucoma drugs. Although cost is an important factor, clearly it should not be the only consideration in choosing a specific medication. Factors to be considered include efficacy of IOP reduction, cost to the patient, cost effectiveness, product shelf life, persistency, and tolerability. Ultimately, choosing the best drug for an individual patient is a decision to be made between physician and patient.

## Abbreviations

AWP = average wholesale price

IOP = intraocular pressure

mm Hg = millimeters of mercury

mL = milliliter

## Competing interests

The author(s) declare that they have no competing interests.

## Authors' contributions

MF and AT found pricing and calculated costs. RF designed the study and participated in drafting the manuscript. All authors read and approved the final manuscript.

## Pre-publication history

The pre-publication history for this paper can be accessed here:


